# Regeneration of Functional Neurons After Spinal Cord Injury via *in situ* NeuroD1-Mediated Astrocyte-to-Neuron Conversion

**DOI:** 10.3389/fcell.2020.591883

**Published:** 2020-12-16

**Authors:** Brendan Puls, Yan Ding, Fengyu Zhang, Mengjie Pan, Zhuofan Lei, Zifei Pei, Mei Jiang, Yuting Bai, Cody Forsyth, Morgan Metzger, Tanvi Rana, Lei Zhang, Xiaoyun Ding, Matthew Keefe, Alice Cai, Austin Redilla, Michael Lai, Kevin He, Hedong Li, Gong Chen

**Affiliations:** ^1^Department of Biology, Huck Institutes of Life Sciences, The Pennsylvania State University, University Park, PA, United States; ^2^Department of Neuroscience & Regenerative Medicine, Medical College of Georgia at Augusta University, Augusta, GA, United States; ^3^Guangdong-Hong Kong-Macau Institute of CNS Regeneration, Jinan University, Guangzhou, China

**Keywords:** spinal cord, NeuroD1, astrocyte, neuronal conversion, *in vivo* reprogramming

## Abstract

Spinal cord injury (SCI) often leads to impaired motor and sensory functions, partially because the injury-induced neuronal loss cannot be easily replenished through endogenous mechanisms. *In vivo* neuronal reprogramming has emerged as a novel technology to regenerate neurons from endogenous glial cells by forced expression of neurogenic transcription factors. We have previously demonstrated successful astrocyte-to-neuron conversion in mouse brains with injury or Alzheimer's disease by overexpressing a single neural transcription factor NeuroD1. Here we demonstrate regeneration of spinal cord neurons from reactive astrocytes after SCI through AAV NeuroD1-based gene therapy. We find that NeuroD1 converts reactive astrocytes into neurons in the dorsal horn of stab-injured spinal cord with high efficiency (~95%). Interestingly, NeuroD1-converted neurons in the dorsal horn mostly acquire glutamatergic neuronal subtype, expressing spinal cord-specific markers such as Tlx3 but not brain-specific markers such as Tbr1, suggesting that the astrocytic lineage and local microenvironment affect the cell fate after conversion. Electrophysiological recordings show that the NeuroD1-converted neurons can functionally mature and integrate into local spinal cord circuitry by displaying repetitive action potentials and spontaneous synaptic responses. We further show that NeuroD1-mediated neuronal conversion can occur in the contusive SCI model with a long delay after injury, allowing future studies to further evaluate this *in vivo* reprogramming technology for functional recovery after SCI. In conclusion, this study may suggest a paradigm shift from classical axonal regeneration to neuronal regeneration for spinal cord repair, using *in vivo* astrocyte-to-neuron conversion technology to regenerate functional new neurons in the gray matter.

## Introduction

Spinal cord injury (SCI) is a devastating central nervous system (CNS) disorder and often leads to loss of motor and sensory functions below the injury site, even paralysis depending on the severity of the injury (Adams and Hicks, 2005). The pathophysiological process after SCI is rather complex, resulting in neuronal loss, neuroinflammation, demyelination, and Wallerian degeneration of the axons (Norenberg et al., 2004). Reactive astrogliosis is common to CNS injury, and particularly severe after SCI. Resident astrocytes react to injury-induced cytokines and dramatically upregulate the expression of a number of proteins such as the astrocytic marker GFAP and the neural progenitor markers Nestin and Vimentin (Sofroniew, 2009). These reactive astrocytes also become proliferative and hypertrophic in cell morphology. In the acute phase of SCI, reactive astrocytes play important roles in repairing the blood-spinal cord barrier and restricting the size of the primary injury (Okada et al., 2006; Herrmann et al., 2008). However, in the sub-acute or chronic phase, reactive astrocytes constitute the major component of the glial scar, a dense tissue structure that is inhibitory to axonal regeneration (Silver and Miller, 2004). Therefore, for decades, substantial effort has been made to overcome the glial scar and promote regrowth of severed axons through the injury site (Filous and Schwab, 2017). On the other hand, the spinal neurons that are lost during and after the injury need to be replaced in order to rebuild the local neuronal circuits. In this regard, stem cell transplantation has been reported to achieve certain success (Tuszynski et al., 2014; Lu et al., 2017), however the identity of transplanted cells and restoration of functional circuitry in the injury site still require further investigation (Goldman, 2016).

*In vivo* neuronal reprogramming has recently emerged as a novel technology to regenerate functional new neurons from endogenous glial cells by overexpressing neurogenic transcription factors in the CNS (Grande et al., 2013; Niu et al., 2013; Guo et al., 2014; Liu et al., 2015, 2020; Gascon et al., 2016; Li and Chen, 2016; Barker et al., 2018; Lei et al., 2019; Chen et al., 2020; Wu et al., 2020). In the injured spinal cord, a combination of growth factor treatment and forced expression of the neurogenic transcription factor *Neurogenin 2* (*Ngn2*) has been reported to stimulate neurogenesis from neural progenitors (Ohori et al., 2006). However, these newly generated neurons suffer from poor long-term survival. More recently, the transcription factor *Sox2* has been shown to reprogram astrocytes into proliferating neuroblasts, which require further treatment with a histone deacetylase inhibitor, valproic acid (VPA), to differentiate into mature neurons (Su et al., 2014). With additional treatment of neurotrophic factors, *Sox2*-converted neurons can express several neuronal subtype markers, but predominately VGluT, a marker for glutamatergic neurons (Wang et al., 2016).

Our group has previously demonstrated that the neurogenic transcription factor *NeuroD1* can reprogram reactive astrocytes into functional neurons in the stab-injured brain and in a mouse model for Alzheimer's disease (Guo et al., 2014). Following studies demonstrated that NeuroD1-mediated *in vivo* astrocyte-to-neuron conversion can reverse glial scar back to neural tissue (Zhang et al., 2020) and repair the damaged motor cortex after ischemic stroke (Chen et al., 2020). More recently, by combining NeuroD1 and Dlx2 together, we have demonstrated that astrocytes in the striatum of R6/2 mouse model for Huntington's disease can be converted into GABAergic neurons (Wu et al., 2020). The major goal of the current study is to determine whether *NeuroD1* can reprogram reactive astrocytes into functional neurons in the injured spinal cord. Using adeno-associated virus (AAV) for gene delivery and a *Cre-Flex* system with *GFAP* promoter to target reactive astrocytes specifically, our results indicate that NeuroD1 can mediate direct astrocyte-to-neuron conversion with high efficiency in both stab and contusive SCI models. The NeuroD1-converted neurons preferentially acquire glutamatergic phenotype in the dorsal horn and express neuronal subtype markers specific to the spinal cord such as Tlx3. Patch clamp recordings further demonstrate that the NeuroD1-converted neurons can functionally mature and integrate into spinal cord circuitry. Interestingly, combining NeuroD1 together with Dlx2 also generates more GABAergic neurons in the spinal cord, similar to that discovered in the brain. Together, our results indicate that NeuroD1-mediated neuronal conversion opens an avenue to treat SCI with internal glial cells through AAV-based gene therapy that may regenerate a diversity of neuronal subtypes for functional repair.

## Materials and Methods

### Animal Use

GAD-GFP mice (Tg[Gad1-EGFP]94Agmo/J) and wild-type C57BL/6 mice were purchased from the Jackson Laboratory and bred in house. Mice of 2–4 months old (both male and female) were used. Mice were housed in a 12 h light/dark cycle and supplied with sufficient food and water. All animal use and studies were approved by the Institutional Animal Care and Use Committee (IACUC) of the Pennsylvania State University. All procedures were carried out in accordance with the approved protocols and guidelines of National Institute of Health (NIH).

### Retrovirus and AAV Production

Retroviral vectors expressing *GFP* and *NeuroD1-GFP* under the *CAG* promoter (*pCAG*) were previously described (Guo et al., 2014). Retrovirus packaging, purification and titering were performed as previously described (Guo et al., 2014). The viral titers were determined by serial dilution to be ~1 × 10^7^ genome copy (GC)/ml.

For AAV-mediated gene expression, the *Cre-Flex* system was applied to target transgene expression specifically to reactive astrocytes using the *human GFAP* (*hGFAP*) promoter. To generate *pAAV-hGFAP::Cre* vector, the *hGFAP* promoter was first amplified from *pDRIVE-hGFAP* plasmid (InvivoGen) by PCR and inserted into *pAAV-MCS* (Cell Biolab) between the MluI and SacII sites to replace the *CMV* promoter. The *Cre* gene coding fragment was then similarly subcloned from *phGFAP-Cre* (Addgene plasmid #40591) and inserted into *pAAV MCS* between the EcoRI and SalI sites. To construct *pAAV-FLEX* vectors expressing transgenes, the coding sequences of *NeuroD1, mCherry* or *GFP* were amplified by PCR from the corresponding retroviral constructs. The *NeuroD1* fragment was fused with either *P2A-mCherry* or *P2A-GFP* and subcloned into the *pAAV-FLEX-GFP* vector (Addgene plasmid #28304) between the KpnI and XhoI sites. All plasmid constructs were confirmed by sequencing. The AAV-CamKII-GFP plasmid was purchased from Addgene (#64545). For AAV production, HEK 293T cells were transfected with the *pAAV* expression vectors, *pAAV9-RC* vector (Cell Biolab), and *pHelper* vector (Cell Biolab) to generate AAV particles carrying our transgenes. Three days after transfection, the cells were scraped in their medium and centrifuged. The supernatant was then discarded and the cell pellet was frozen and thawed four times, resuspended in a discontinuous iodixanol gradient, and centrifuged at 54,000 rpm for 2 h. Finally, the virus-containing layer was extracted, and the viruses were concentrated using Millipore Amicon Ultra Centrifugal Filters. The viral titers were determined using the QuickTiter™ AAV Quantitation Kit (Cell Biolabs) and then diluted to a final concentration of 1 × 10^10^ GC/ml for injection (except for our GFAP-Cre virus, diluted to a final concentration of 1 × 10^8^ GC/ml).

### Laminectomy, Injury, and Stereotaxic Viral Injection

Mice were anesthetized by intraperitoneal injection of ketamine/xylazine (80–120 mg/kg ketamine; 10–16 mg/kg xylazine). A laminectomy was then performed at the T11–T12 vertebrae to expose dorsal surface of the spinal cord, and a stab or contusion injury was performed. The stab injury was conducted with a 31-gauge needle at the center of the exposed surface, 0.4 mm lateral to the central artery with a depth of 0.4 mm, while the contusion injury was generated with a force of 45 kdyn on an Infinite Horizon Impactor (IH-0400, Precision Systems and Instrumentation) directly at the center of the exposed surface. For conversion after stab injury, 1.0 μL of AAV (1 × 10^10^ GC/ml) was injected using a 50 μl Hamilton syringe with a 34-gauge injection needle at a rate of 0.05 μL/min at the same coordinates immediately after the stab injury, while retrovirus (1 × 10^7^ GC/ml) was injected at 4 days post stab injury. For conversion after contusive injury, AAV (1 × 10^10^ GC/ml) was injected at 10 days or 16 weeks following contusion injury and at 1 mm away from the injury site with a depth of 0.4–0.8 mm. The viral injection needle was kept in place for 3 min after injection to prevent drawing out the virus during withdrawal. The surgical area was then treated with antibiotic ointment and the skin was clipped for a week to allow the skin to re-suture. The mice were kept on a heating pad and treated with Carprofen for pain relieve via subcutaneous injection (5 mg/kg) on the day of surgery and drinking water (10 mg/kg) for 3 days after surgery and closely monitored for 1 week to ensure full recovery of health.

### Electrophysiology

Mice were sacrificed at defined time points by anesthetization with 2.5% Avertin and decapitation. The spinal cord segment was then removed from the spine into cutting solution (125 mM NaCl, 2.5 mM KCl, 1.3 mM MgSO_4_, 26 mM NaHCO_3_, 1.25 mM NaH_2_PO_4_, 2.0 mM CaCl_2_, and 10 mM glucose adjusted to pH 7.4 and 295 mOsm/L and bubbled for 1 h with 95% O_2_/5% CO_2_) cooled on ice, where it was encased in an agarose matrix (Sigma) and cut into 300 μm thickness slices using a VT3000 vibratome (Leica). Slices were then incubated for 1 h in holding solution (92 mM NaCl, 2.5 mM KCl, 1.25 mM NaH_2_PO_4_, 30 mM NaHCO_3_, 20 mM HEPES, 15 mM glucose, 12 mM N-Acetyl-L-cysteine, 5 mM Sodium ascorbate, 2 mM Thiourea, 3 mM Sodium pyruvate, 2 mM MgSO_4_, and 2 mM CaCl_2_, adjusted to pH 7.4 and 295 mOsm/L and bubbled continuously with 95% O_2_/5% CO_2_) at room temperature before patch-clamp recording in standard ACSF (125 mM NaCl, 2.5 mM KCl, 1.25 mM NaH_2_PO_4_, 26 mM NaHCO_3_, 1.3 MgSO_4_, 2.5 mM CaCl_2_, and 10 mM glucose adjusted to pH 7.4 and 295 mOsm/L and bubbled for 1 h with 95% O_2_/5% CO_2_). Both native and converted cells were recorded by whole-cell recording using standard inner solution (135 mM K-gluconate, 10 mM KCl, 5 mM Na-phosphocreatine, 10 mM HEPES, 2 mM EGTA, 4 mM MgATP, and 0.5 mM Na_2_GTP, adjusted to pH 7.4 and 295 mOsm/L) with the membrane potential held at −70 mV. Typical values for the pipette and total series resistances were 2–10 and 20–60 MΩ, respectively. Data were collected using the pClamp 9 software (Molecular Devices) by sampling at 10 kHz and filtering at 1 kHz. Data were then analyzed and plotted with the Clampfit 9.0 software (Molecular Devices).

### Immunohistochemistry, Immunocytochemistry, and Microscopy

After perfusion, the target region of the spinal cord (~0.5 cm in length) was surgically dissected, fixed in 4% paraformaldehyde (PFA) in PBS for 1 day, dehydrated in 30% sucrose solution for 1 day, and sectioned into 30 μm coronal or horizontal slices using a Leica CM1950 cryostat. The slices were collected serially in 24-well plates so that distance from the injury site could later be ascertained. The samples were then stored at 4°C in 0.02% sodium azide (NaN_3_) in PBS to prevent bacterial degradation. Spinal cord slices were chosen for immunohistochemistry based on infection of the dorsal horn by inspecting the reporter protein (mCherry or GFP) in the storage solution under a fluorescent microscope. For the stab injury experiments, care was taken to select coronal slices at least 100 μm from the injury site to ensure tissue integrity. On the first day of staining, samples were washed in PBS three times for 5 min per wash, permeablized with 2% Triton X-100 in PBS for 20 min, and blocked using a 5% normal donkey serum (NDS) and 0.1% Triton-X in PBS for 2 h to reduce non-specific binding of the antibodies. The samples were then incubated with primary antibodies diluted in the same blocking buffer at 4°C for two nights to allow thorough penetration of the antibodies. On the third day, the samples were recovered to room temperature, washed in PBS three times for 5 min per wash, and incubated with secondary antibodies diluted in blocking buffer for 1 h. Finally, the samples were washed in PBS three more times for 10 min per wash and mounted on glass slides with coverslips using anti-fading mounting solution (Invitrogen). The immunostained samples were examined and imaged using Olympus FV1200 and Zeiss LSM 800 laser confocal microscopes. Z-stacks were collected for the *in vivo* images for the whole thickness of the samples and maximum intensity and z-stack projections were used for image preparation and analysis.

### Quantification and Data Analysis

As a result of our carefully selected injection coordinates described above, infected cells were mostly found in the dorsal horn of the spinal cord, Rexed laminae 1-6 (Rexed, 1954). For most of the quantification, including cell conversion and NeuN acquisition, cells were counted if they appeared in any part of this region. For quantification based on cell subtype (**Figures 3, 4**), cells were only counted if they appeared in Rexed laminae 1-3, centered about the substantia gelatinosa, a region dominated by small, excitatory interneurons and easily demarcated due to its high cell density (Santos et al., 2007). This region was chosen for its ease of demarcation and so that a consistent local population of neuronal subtypes could be expected from sample to sample. Quantification was performed on collected images using the z-stacked images as a guide and the layered stacks to check the vertical dimension. Strict background cutoffs for positive signals were calculated for each channel as three times the average background intensity for the relevant tissue and antibody. Cells were binned by presence (i.e., above the background cutoff) or absence (i.e., below the background cutoff) for each marker in question, using the viral fluorophore (mCherry or GFP) to identify infected cells and DAPI to confirm each cell for counting. To estimate the total number of converted neurons per infection for our contusion experiments, we multiplied the average number of NeuN^+^ neurons among the infected cells per horizontal section, calculated from one dorsal, one central, and one ventral section, by the total number of horizontal sections per sample. While this might over estimate the number of cells, it gives a rough estimate of the number of converted neurons in the infected areas. All quantification was performed on three biological replicates per data point and is reported as the means and standard deviations of the three replicates.

## Results

### NeuroD1 Reprograms Reactive Astrocytes Into Neurons in the Injured Spinal Cord

**Figure 1:**
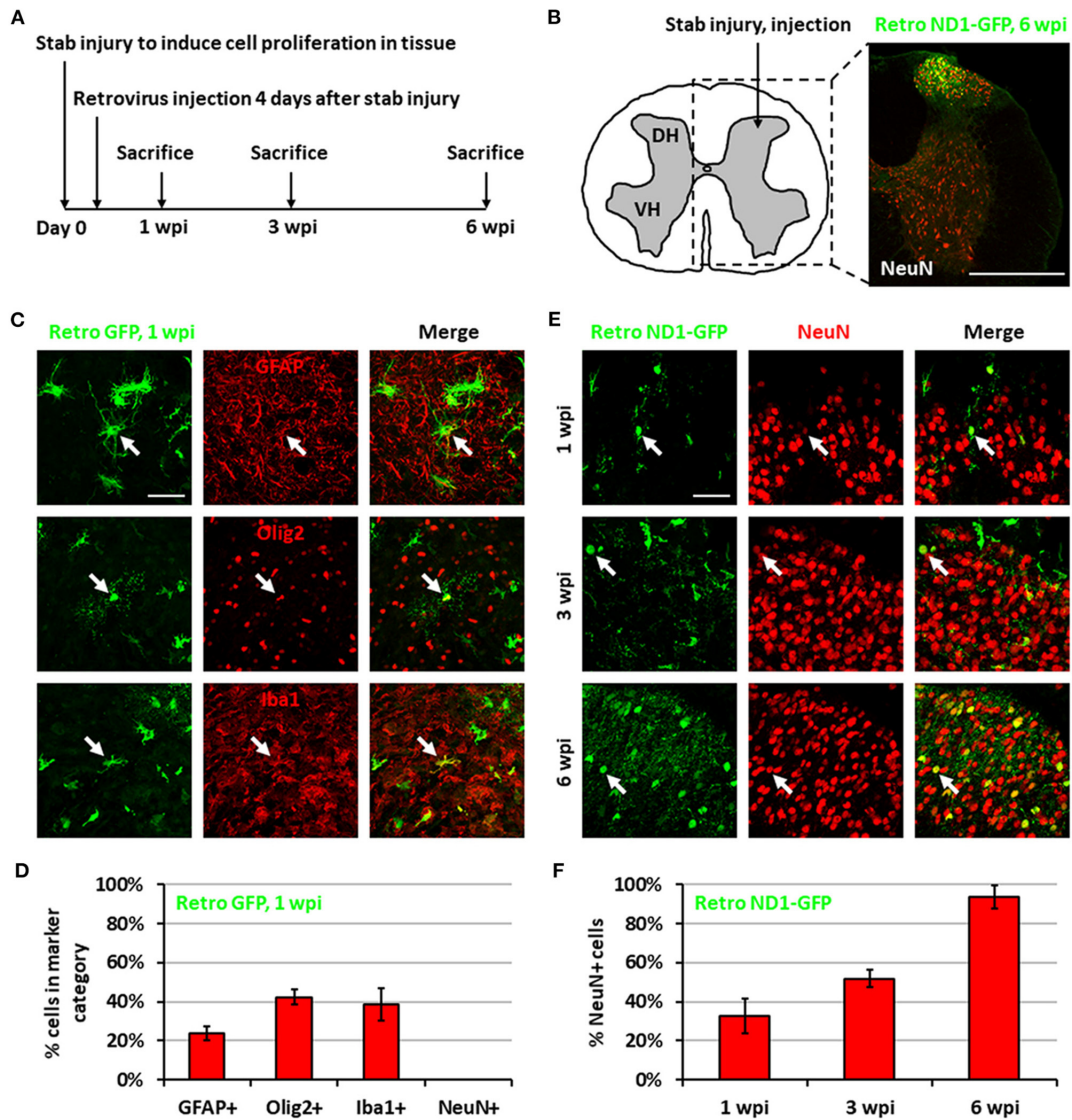
Neuronal conversion induced by NeuroD1-expressing retroviruses after stab injury in the spinal cord dorsal horn. **(A)** Experiment paradigm. **(B)** Schematic illustration of dorsal horn injury and viral injection, together with an actual viral infection image. Coordinates are 0.4 mm lateral of the central artery and 0.4 mm below the tissue surface. Stab injury was performed with a 32-guage needle followed by stereotaxic injection at the injury site. Scale bar, 500 μm. **(C)** Three main types of proliferating glial cells at 1 wpi after injection of control retrovirus expressing GFP alone: astrocytes (GFAP), OPCs (Olig2), and microglia (Iba1). Scale bar, 50 μm. **(D)** Quantification based on staining for control retrovirus GFP at 1 wpi. Bars show the mean and standard deviation of three replicates. **(E)** NeuroD1-GFP-infected cells in the dorsal horn at 1, 3, and 6 wpi after retroviruses expressing NeuroD1-GFP. NeuroD1-GFP-infected cells gradually acquired more and more NeuN signal. Arrows show example NeuN^+^ cells. Scale bar, 500 μm. **(F)** Quantification based on staining for retroviruses expressing NeuroD1-GFP. Bars show the mean and standard deviation of three replicates.

We previously demonstrated that expressing NeuroD1 in reactive astrocytes after brain injury can directly convert astrocytes into neurons (Guo et al., 2014; Chen et al., 2020; Zhang et al., 2020). In this study, we investigated whether such *in vivo* direct conversion technology can regenerate functional new neurons in injured spinal cord. To target the dividing reactive astrocytes after injury, we employed retroviruses that mainly express ectopic genes in dividing cells but not in neurons, which cannot divide. We injected NeuroD1-expressing retroviruses at 4 days post-stab injury (dpi), when many dividing reactive astrocytes have been detected (Chen et al., 2008; Hong et al., 2014), and analyzed samples at 1, 3, and 6 weeks post-injection (wpi) (Figure 1A). In this study, we chose the spinal cord dorsal horn as our major region of interest because it is composed of both excitatory and inhibitory neurons and is critical to afferent sensory information processing (Figure 1B). We are currently investigating motor neuron regeneration in the spinal cord ventral horn in a separate study. We first explored the cell types infected by our control CAG::GFP retroviruses. At 1 wpi, we found that the control GFP retroviruses infected a mixture of glial cell types including reactive astrocytes (GFAP^+^ and some GFAP^+^/Olig2^+^), oligodendrocyte progenitor cells (OPCs) (Olig2^+^), and microglia (Iba1^+^) (Figures 1C,D), but not NeuN^+^ neurons (Figure 1D). In contrast, cells infected by the CAG::NeuroD1-GFP retrovirus showed an increasing number of NeuN^+^ cells with neuronal morphology over time (Figure 1E), and quantitatively reached 93.5% at 6 wpi (Figure 1F), indicating a successful glia-to-neuron conversion in the injured spinal cord.

**Figure 2:**
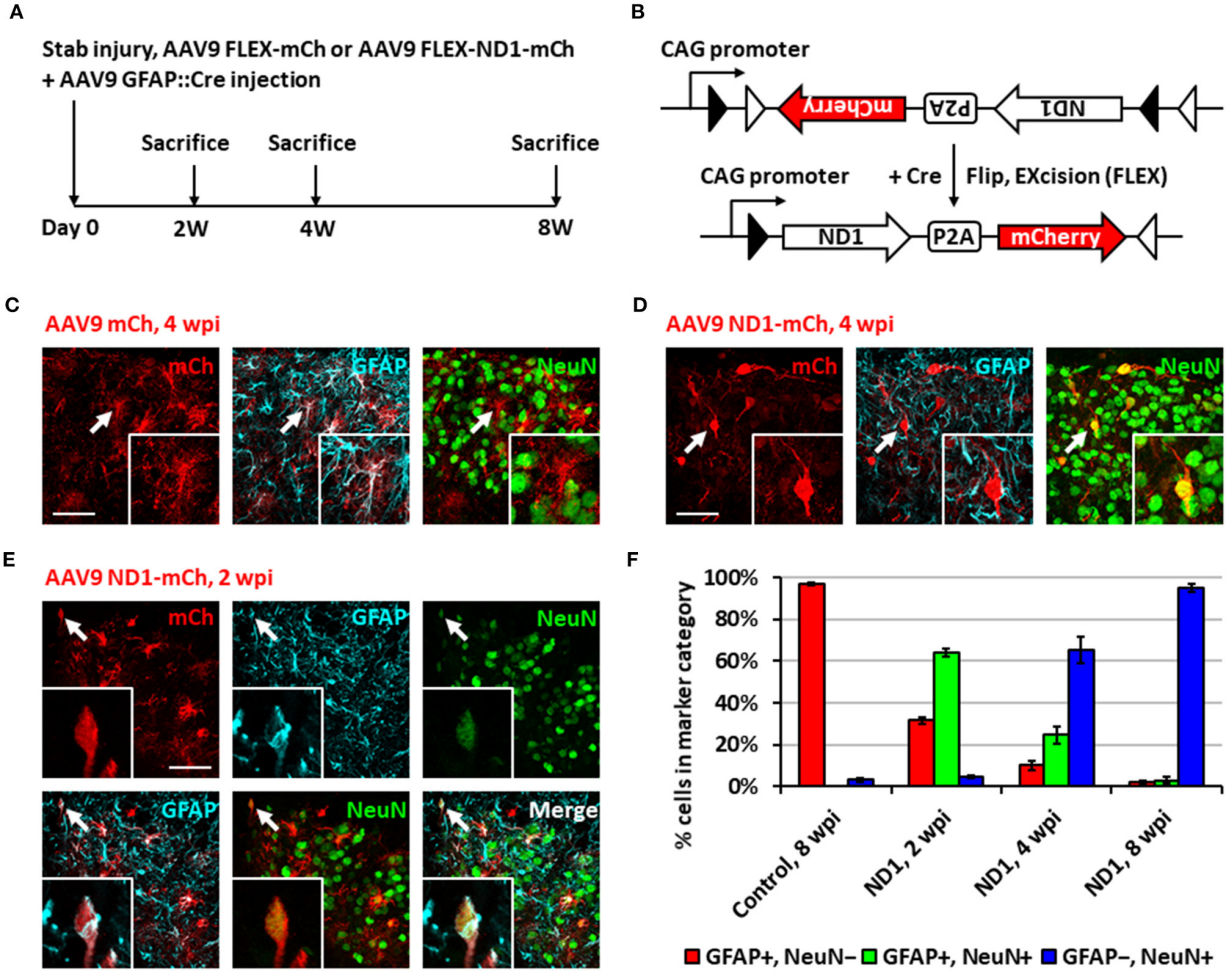
Neuronal conversion using AAV9 NeuroD1 viruses after stab injury in the spinal cord dorsal horn. **(A)** Experiment paradigm. **(B)** AAV9 GFAP::Cre and AAV9 FLEX-NeuroD1-mCherry system (abbreviated elsewhere as AAV9 ND1-mCh). GFAP promoter restricts infected cells to astrocytes. Control virus replaces the NeuroD1 transgene with an additional mCherry. **(C)** Infected astrocytes in the dorsal horn at 4 wpi after control AAV9 mCherry injection. Arrows and inset show an example of mCherry-infected GFAP^+^ astrocyte. Scale bar, 50 μm. **(D)** AAV9 NeuroD1-mCherry-infected cells became converted neurons in the dorsal horn at 4 wpi after viral injection. Arrows and inset show an example of NeuroD1-mCherry-labeled NeuN^+^ cell. Scale bar, 50 μm. **(E)** A transitional cell (arrows and inset) captured during astrocyte-to-neuron conversion process that shows both GFAP^+^ and NeuN^+^ signals at early time point of 2 weeks after NeuroD1-mCherry infection. Scale bar, 50 μm. **(F)** Quantification data based on GFAP and NeuN staining for control or NeuroD1-infected cells at different time points (2, 4, and 8 wpi). Bars show the mean and standard deviation of three replicates. Infected cells at 2 wpi are mostly transitional, staining positive for both NeuN and GFAP. By 4 wpi, NeuroD1-infected cells are mostly converted neurons, staining positive for only NeuN.

While retrovirus has the advantage to target only dividing glial cells for conversion, avoiding non-dividing neurons, such advantage also limits its capability to convert non-dividing glial cells into neurons. In order to move our *in vivo* reprogramming technology toward clinical settings in the future, we adopted an AAV gene delivery system in which the transgene expression is controlled by an astrocyte-specific *GFAP* promoter to target both dividing and non-dividing glial cells (Figure 2A). Specifically, we used a *Cre-Flex* gene expression system, which contains two AAV vectors, with one encoding *GFAP-Cre* and the other encoding the transgene in reverse form flanked by double LoxP sites (FLEX vector) (Atasoy et al., 2008; Liu et al., 2015; Chen et al., 2020). Thus, when the two AAVs are co-injected into the spinal cord, Cre recombinase will be expressed in the infected reactive astrocytes and turn on the transgene expression in FLEX vector by flipping the transgene sequence into the correct form for transcription (Figure 2B). We first confirmed the specificity of the *Cre-Flex* AAV system in the spinal cord by co-injecting AAV GFAP::Cre and AAV FLEX-CAG::mCherry (or::GFP) into the stab-injured dorsal horn. The control virus infected cells were mostly GFAP^+^, NeuN^−^ astrocytes at 4 wpi (Figure 2C). Next, we co-injected AAV GFAP::Cre with AAV FLEX-CAG::NeuroD1-P2A-mCherry into the stab-injured dorsal horn. In contrast to the control AAV, the NeuroD1-mCherry infected cells were mostly NeuN^+^/GFAP^−^ neurons with clear neuronal morphology at 4 wpi (Figure 2D). NeuroD1 overexpression in the infected cells was confirmed by immunostaining (Supplementary Figure 1). Interestingly, besides NeuN^+^/GFAP^−^ converted neurons, we also observed many NeuroD1-AAV-infected cells at 2 wpi with co-immunostaining of both GFAP^+^ and NeuN^+^ (Figure 2E), suggesting a potential intermediate stage during astrocyte-to-neuron conversion. We termed these GFAP^+^/NeuN^+^ cells induced by NeuroD1 expression in astrocytes as “AtN transitional cells.” We did not observe any such transitional cells in the control mCherry-infected spinal cord after injury, suggesting that AtN conversion does not happen following neural injury but can be induced by ectopic expression of transcription factors such as NeuroD1. Quantitative analysis revealed that the control AAV-infected cells were mostly GFAP^+^ astrocytes by 8 wpi (Figure 2F, left red bar), whereas NeuroD1 AAV-infected cells showed a progressive increase in the percentage of neurons (NeuN^+^/GFAP^−^, blue bar in Figure 2F) from 2 to 8 wpi, reaching ~95% at 8 wpi (Figure 2F, right blue bar). Note that at 2 wpi, over 60% of NeuroD1-infected cells were GFAP^+^/NeuN^+^ transitional cells (green bar in Figure 2F), which gradually decreased at 4 wpi and 8 wpi together with a decrease of GFAP^+^ astrocytes (red bar in Figure 2F) among the NeuroD1-infected cell population. Further analysis showed that neither transitional cells nor converted neurons exhibited significant cell death suggesting that apoptosis does not play a significant role during the NeuroD1-mediated cell conversion process (Supplementary Figure 2). Comparing to Ngn2-mediated or Ascl1-mediated AtN conversion (Gascon et al., 2016), less apoptosis was detected during NeuroD1-mediated conversion process, which may suggest that different transcription factors act through different signaling and metabolic pathways to carry out cell conversion.

### NeuroD1 Converts Dorsal Spinal Astrocytes Into Tlx3^+^ Glutamatergic Neurons

**Figure 3:**
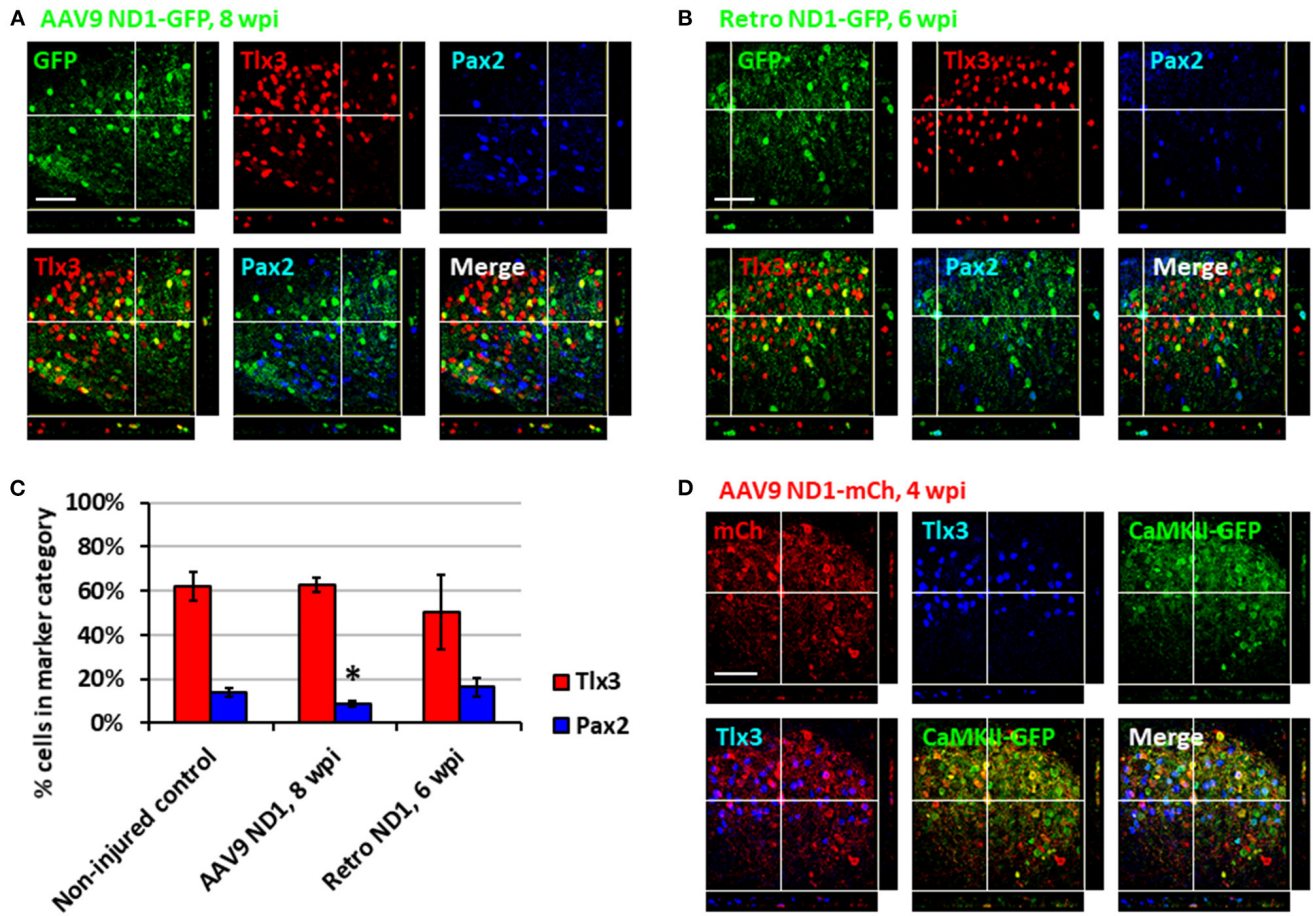
Subtypes of NeuroD1-converted neurons in the spinal cord dorsal horn. **(A)** Tlx3 (glutamatergic) and Pax2 (GABAergic) subtype staining for converted neurons in the dorsal horn at 8 wpi after AAV9 NeuroD1-GFP injection. Z-projection targets an example of Tlx3^+^ neuron. Scale bar, 50 μm. **(B)** Tlx3 and Pax2 subtype staining for converted neurons in the dorsal horn 6 wpi after retrovirus ND1-GFP injection. Z-projection targets an example of Pax2^+^ neuron. Scale bar, 50 μm. **(C)** Quantification based on subtype staining after AAV9 ND1-GFP (8 wpi) or retrovirus ND1-GFP (6 wpi) conversion. Control data is based on NeuN^+^ cells in uninjured, untreated tissue. Bars show the mean and standard deviation of three replicates. Kruskal-Wallis *H*-tests show no significant difference in % of Tlx3^+^ or Pax2^+^ neurons between the non-injured control and the retrovirus ND1-GFP groups (*p* > 0.05). Kruskal-Wallis *H*-tests show no significant difference in % of Tlx3^+^ neurons (*p* > 0.05) but a significant difference in % of Pax2^+^ neurons between the non-injured control and the AAV9 ND1-GFP groups (*p < 0.05). **(D)** AAV9 ND1-mCherry and CaMK2-GFP co-injection showed strong (89.5 ± 5.2%; *n* = 3) co-labeling of CaMK2 for converted Tlx3^+^ neurons at 4 wpi. Z-projection targets an example of Tlx3^+^, CaMK2^+^ neuron. Scale bar, 50 μm.

After demonstrating astrocyte-to-neuron conversion in the spinal cord, we next investigated which subtypes of neurons were generated through NeuroD1-mediated conversion. The dorsal horn of the spinal cord contains two main neuronal subtypes: glutamatergic and GABAergic neurons (Abraira and Ginty, 2013). During spinal cord development, two transcription factors, Tlx3 and Pax2, appear to play critical roles in determining cell fate specification in the dorsal horn (Cheng et al., 2005; Huang et al., 2008). Interestingly, by examining AAV NeuroD1-GFP-infected cells in the dorsal horn at 8 wpi, we found that the majority of NeuroD1-converted neurons were Tlx3^+^ (62.6 ± 3.3%), suggesting a majority glutamatergic neuronal subtype (Figure 3A). In contrast, only a small percentage of NeuroD1-converted neurons in the dorsal horn were Pax2^+^ (8.8 ± 1.3%), suggesting a minority GABAergic neuronal subtype (Figure 3A). Because AAV might infect a small proportion of neurons (Figure 2F, control), we further examined retrovirus NeuroD1-GFP-infected cells in the dorsal horn at 6 wpi and found that, similar to our AAV experiments, the retrovirus NeuroD1-converted neurons were mainly Tlx3^+^ (50.3 ± 17.0%), with a minority being Pax2^+^ (16.4 ± 4.3%) (Figure 3B; quantified in Figure 3C). These results suggest that the majority of NeuroD1-converted neurons in the dorsal horn of spinal cord are Tlx3^+^ neurons, with a small proportion being Pax2^+^ neurons.

We further confirmed the neuronal subtypes after NeuroD1 conversion using AAV CaMKII-GFP to identify glutamatergic neurons and GAD-GFP transgenic mice to identify GABAergic neurons. When co-injecting AAV GFAP::Cre and Flex-NeuroD1-mCherry together with AAV CaMKII::GFP (Dittgen et al., 2004), we observed 89.5 ± 5.2% (*n* = 3) of GFP^+^ cells co-expressing Tlx3^+^, confirming that these Tlx3^+^ neurons are indeed glutamatergic (Figure 3D). Many NeuroD1-mCherry converted neurons were also colocalizing with CaMKII-GFP (Figure 3D), suggesting that they were glutamatergic neurons. When injecting AAV GFAP::Cre and Flex-NeuroD1-mCherry in GAD-GFP mice (Ma et al., 2006), in which GABAergic neurons are genetically labeled with GFP, we did not observe GAD-GFP co-expression with Tlx3^+^ (*n* = 3), as expected. Indeed, in the dorsal horn of spinal cord in uninjured and untreated mice, we observed that the CaMKII-GFP co-stained consistently with endogenous Tlx3^+^ while GAD-GFP co-stained with Pax2^+^ (Supplementary Figure 3). Therefore, the majority of NeuroD1-converted neurons in the dorsal horn of the spinal cord are glutamatergic neurons, consistent with our findings in the mouse cortex (Chen et al., 2020).

### Dlx2 Combined With NeuroD1 Increases the Conversion of Dorsal Spinal Astrocytes Into Pax2^+^ GABAergic Neurons

**Figure 4:**
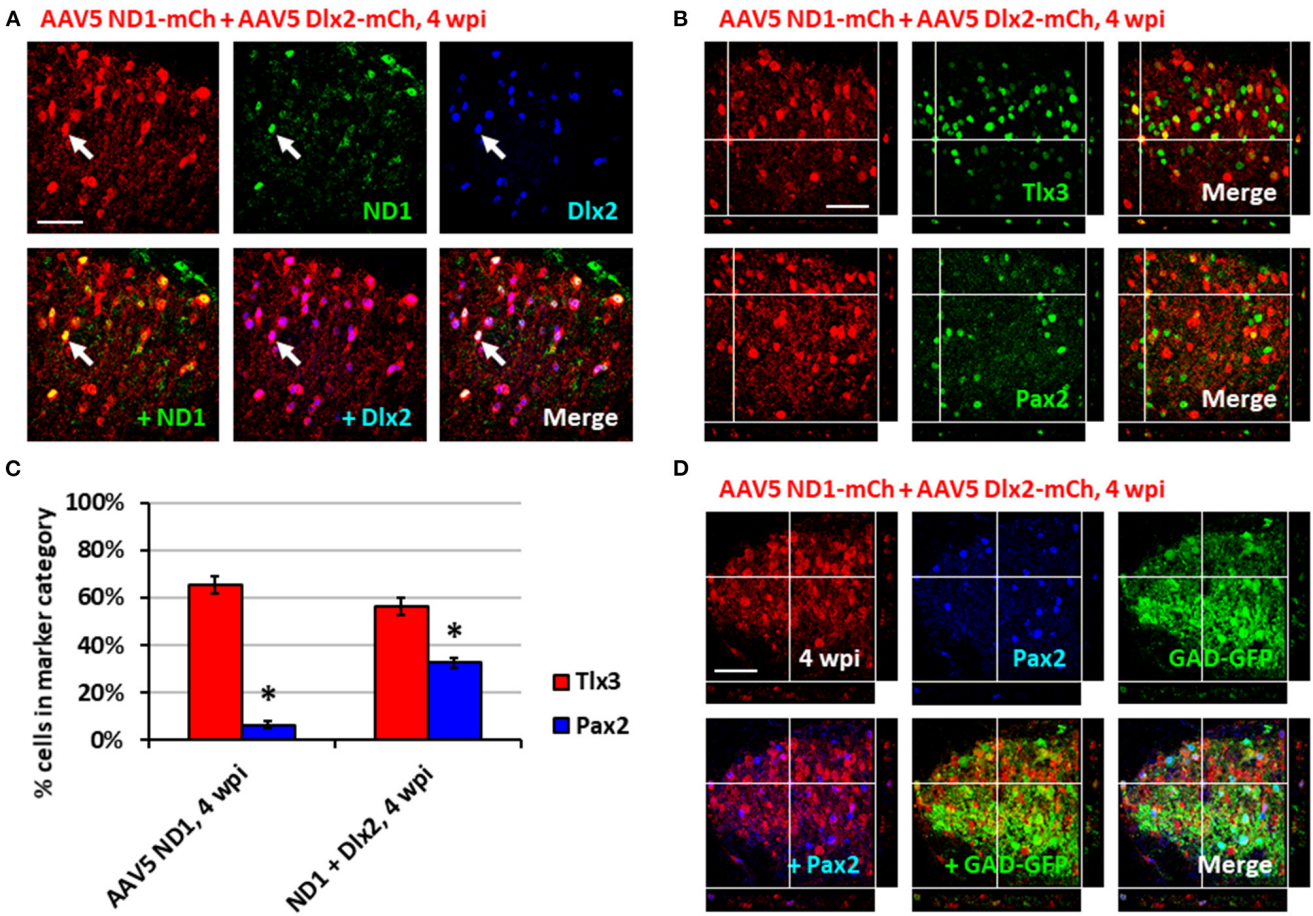
Combining Dlx2 together with NeuroD1 generated more GABAergic neurons in the spinal cord dorsal horn. **(A)** Immunostaining confirmed the overexpression of both NeuroD1 and Dlx2 at 4 wpi following AAV5 NeuroD1-mCherry + AAV5 Dlx2-mCherry infection (1:1 ratio of 1 × 10^10^ GC/ml AAV5 NeuroD1-mCherry and 1 × 10^10^ GC/ml AAV5 Dlx2-mCherry mixed 9:1 with 1 × 10^9^ GC/ml AAV5 GFAP-Cre for a final titer of 5 × 10^9^ GC/ml AAV5 NeuroD1-mCherry, 5 × 10^9^ GC/ml Dlx2-mCherry, 1 × 10^8^ GC/ml AAV5 GFAP-Cre; 1 μL of virus mixture injected per mouse). Scale bar, 50 μm. **(B)** Tlx3 and Pax2 staining for converted neurons in the dorsal horn at 4 wpi after NeuroD1 and Dlx2 infection. Z-projections target example of Tlx3^+^ or Pax2^+^ neurons. Scale bar, 50 μm. **(C)** Quantitative comparison of Tlx3 and Pax2 subtype staining between neurons converted by NeuroD1 alone (4 wpi) or NeuroD1 + Dlx2 (4 wpi). Bars show the mean ± S.D. (*n* = 3 replicates). Kruskal-Wallis *H*-tests show significant differences in % Tlx3^+^ and % Pax2^+^ between the two groups (*p < 0.05). **(D)** Co-immunostaining showing co-localization of NeuroD1+Dlx2-converted neurons (mCherry+) together with Pax2 and GAD-GFP at 4 wpi (*n* = 3). Z-projection targets an example of converted neuron being Pax2^+^ and GAD^+^. Scale bar, 50 μm.

Having generated a majority of glutamatergic neurons using NeuroD1 alone in the spinal cord, we further investigated whether it's possible to increase the proportion of GABAergic neurons by combining NeuroD1 with other transcription factors. In a separate study on Huntington's disease, we have discovered that combining NeuroD1 and Dlx2 together can successfully convert striatal astrocytes into GABAergic neurons (Wu et al., 2020). Dlx2 is a transcription factor that has been reported to play a significant role in GABAergic neuron specification and maturation during brain development (Anderson et al., 1999; McKinsey et al., 2013; Victor et al., 2014; Yang et al., 2017; Pla et al., 2018). Therefore, we injected a 1:1 ratio of AAV5 FLEX-NeuroD1-mCherry and AAV5 FLEX-Dlx2-mCherry in combination with AAV5-GFAP-Cre (Figure 4; *n* = 3). We first confirmed the co-expression of NeuroD1 and Dlx2 with immunostaining after viral infection (Figure 4A). Immunostaining experiments demonstrated that many NeuroD1+Dlx2-converted neurons were Tlx3^+^ or Pax2^+^ neurons (Figure 4B). Quantitative analysis revealed that 32.5 ± 2.1% of NeuroD1+Dlx2-converted neurons were Pax2^+^ neurons (Figure 4C), a 5-fold increase compared to that generated by NeuroD1 alone (6.3%; *p* = 0.05, Kruskal-Wallis *H*-test). The percentage of Tlx3^+^ neurons generated by NeuroD1 + Dlx2 was 56.2 ± 3.4% (Figure 4C). Importantly, the GABAergic identity of our NeuroD1+Dlx2-converted neurons was further confirmed in GAD-GFP mice, where we observed co-localization of NeuroD1+Dlx2-converted neurons were both Pax2^+^ and GAD-GFP^+^ (Figure 4D; *n* = 3; 4 wpi). These results suggest that the ratio of newly converted Tlx3^+^ vs. Pax2^+^ neurons in the dorsal horn of spinal cord can be determined through the combinations of NeuroD1 and Dlx2 transcription factors.

### NeuroD1-Converted Neurons Express Region-Specific Neuronal Subtype Markers

**Figure 5:**
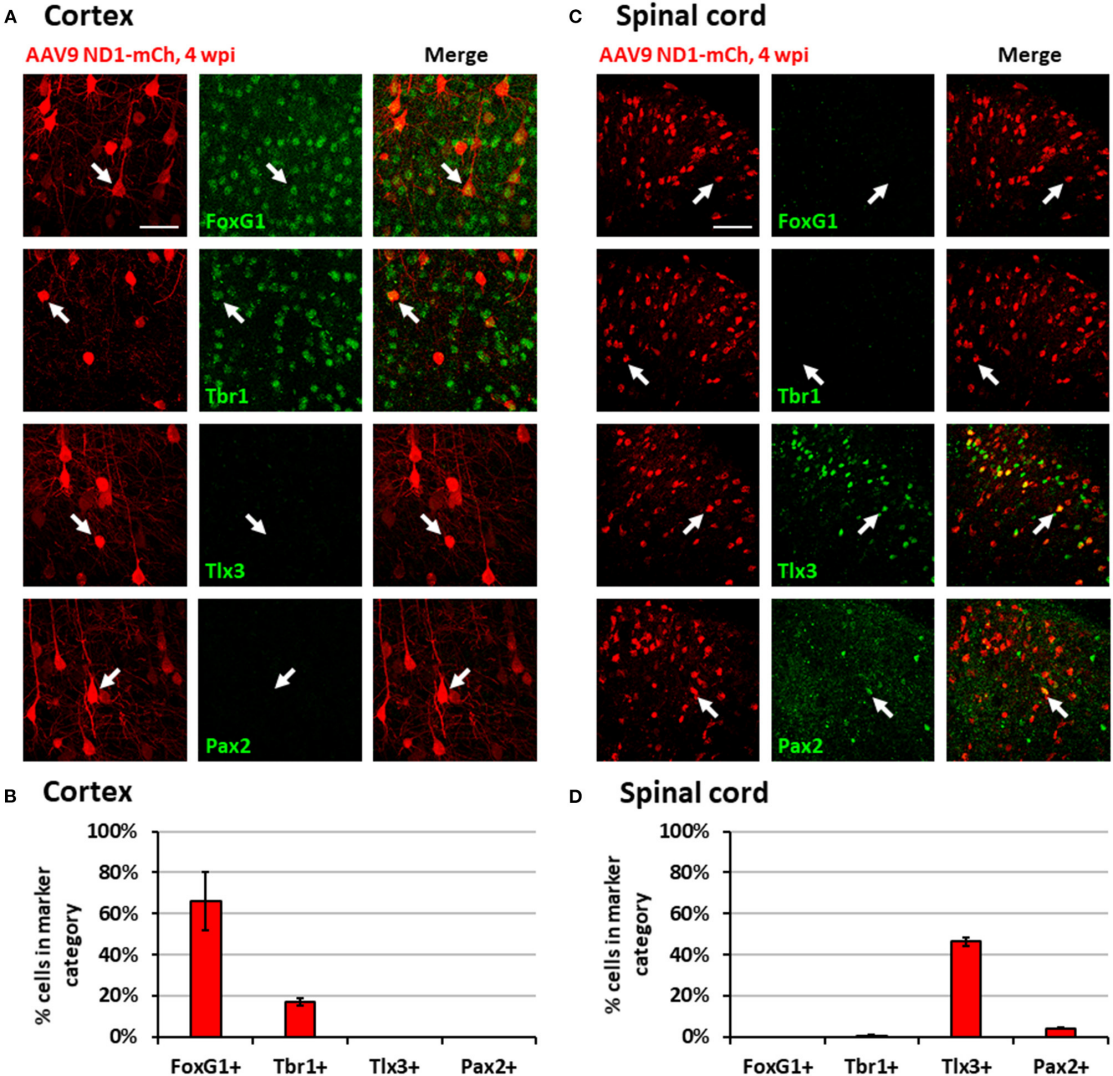
Region-specific subtypes of NeuroD1-converted neurons in the brain vs. the spinal cord. **(A)** Subtype staining for converted neurons in the cortex 4 wpi after AAV9 NeuroD1-mCherry injection. Arrows show examples of cells positive for each subtype. Scale bar, 50 μm. **(B)** Quantification based on subtype staining in the cortex for AAV9 NeuroD1-mCherry samples (4 wpi). Bars show the mean ± S.D. (*n* = 3 replicates). **(C)** Subtype staining for converted neurons in the spinal cord dorsal horn 4 wpi after AAV9 NeuroD1-mCherry injection. Arrows show examples of cells positive for each subtype. Scale bar, 50 μm. **(D)** Quantification based on subtype staining in the spinal cord for AAV9 NeuroD1-mCherry samples (4 wpi). Bars show the mean ± S.D. (*n* = 3 replicates).

While NeuroD1-converted neurons appear to be mainly glutamatergic neurons in both the mouse cortex and spinal cord, we further investigated whether they are the same type of glutamatergic neurons or not. For this purpose, we injected the same AAV GFAP::Cre and AAV FLEX-NeuroD1-mCherry into the mouse M1 motor cortex and the spinal cord, and then performed a serial immunostaining using both cortical neuronal markers (FoxG1 and Tbr1) and spinal neuronal markers (Tlx3 and Pax2) at 4 wpi (Figure 5). The majority of NeuroD1-infected cells were converted into neurons in both the brain and the spinal cord at 4 wpi (Figures 5A,C). Strikingly, when we compared the neuronal subtypes resulting from NeuroD1-mediated conversion in the brain vs. the spinal cord side-by-side, a distinct pattern emerged: the converted neurons in the mouse cortex acquired cortical neuron markers such as FoxG1 (66.1 ± 14.3%) and Tbr1 (17.1 ± 1.9%), but not spinal neuron markers such as Tlx3 (0%) or Pax2 (0%) (Figures 5A,B); in contrast, the converted neurons in the spinal cord acquired spinal neuron markers Tlx3 (46.4 ± 2.2%) and Pax2 (4.2 ± 0.3%), but not cortical neuron markers FoxG1 (0%) or Tbr1 (0.6 ± 0.5%) (Figures 5C,D). Morphologically, the converted neurons in the brain resembled cortical pyramidal neurons with larger cell bodies (Figure 5A), while those in the spinal cord resembled dorsal horn interneurons with smaller cell bodies (Figure 5C). The relative lower percentage of Tbr1^+^ cells among the converted neurons in the cortex suggest that the newly converted neurons may not be mature enough at 4 wpi and may take longer time to fully acquire their neuronal identity. These distinct differences in the neuronal identity after conversion by the same transcription factor in the brain vs. the spinal cord suggest that the glial cell lineage, here cortical lineage vs. spinal lineage, as well as the local environment may exert an important influence on the resulting subtypes of converted neurons.

### NeuroD1-Converted Neurons Are Physiologically Functional

**Figure 6:**
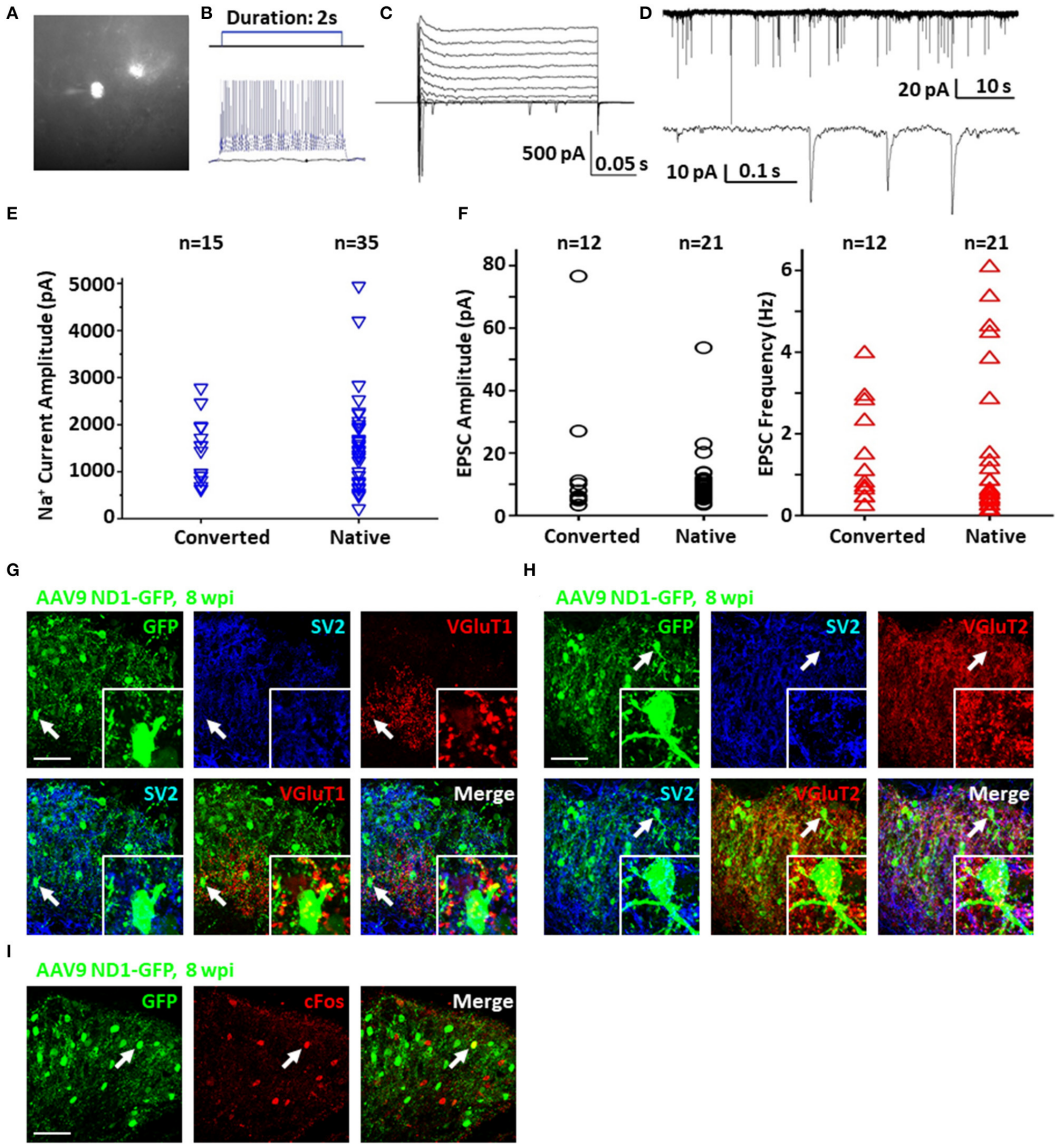
Functionality of NeuroD1-converted neurons in the spinal cord dorsal horn. **(A)** Representative image of a NeuroD1-converted neuron for patch-clamp recording in spinal cord slices. **(B)** Sample action potentials of a converted neuron. **(C)** Sample Na^+^ and K^+^ currents of a converted neuron. **(D)** Sample EPSCs of a converted neuron. **(E)** Na^+^ current amplitudes for converted and native neurons. Student's two-tailed *T*-test shows no significant difference between the two groups (*p* > 0.5). **(F)** EPSC amplitudes and frequencies for converted and native neurons. Student's two-tailed *T*-tests show no significant difference between the two groups (*p* > 0.5). **(G)** Synaptic SV2 and VGluT1 puncta observed on converted neurons in the dorsal horn at 8 wpi after AAV9 NeuroD1-GFP injection. Arrows and inset show puncta on the soma and processes. Scale bar, 50 μm. **(H)** Synaptic SV2 and VGluT2 puncta on converted neurons in the dorsal horn 8 wpi after AAV9 NeuroD1-GFP injection. Arrows and inset show puncta on the soma and processes. Scale bar, 50 μm. **(I)** Integration of converted neurons into local network in the dorsal horn 8 wpi after AAV9 ND1-GFP injection. Activated neurons indicated by c-Fos staining were only a small subset of all neurons. C-Fos staining was performed at 2 h after mouse exercised for 15 min on a running wheel.

To test the functionality and circuit-integration of NeuroD1-converted neurons, we performed patch-clamp electrophysiological recordings of native and converted neurons on spinal cord slices from mice sacrificed at 8–10 wpi (Figure 6A). The converted neurons could generate repetitive action potentials (Figure 6B) and displayed large Na^+^ and K^+^ currents (Figure 6C). Moreover, we detected robust spontaneous EPSCs from the NeuroD1-converted neurons (Figure 6D). Quantitatively, we found that the NeuroD1-converted neurons showed no significant difference in Na^+^ currents (Figure 6E) and spontaneous EPSCs from their neighboring native neurons (Figure 6F). Immunostaining with a series of synaptic markers including SV2 and VGlut1/VGlut2 further confirmed that the NeuroD1-converted neurons were surrounded by numerous synaptic puncta with many of them directly innervating the neuronal soma and dendrites (Figures 6G,H, cyan and yellow dots). Finally, cFos, an immediate early gene that is typically activated by neuronal activity during functional tasks, was clearly detected in some of the NeuroD1-converted neurons, indicating that they were functionally active in the local spinal cord circuits (Figure 6I). Altogether, our results demonstrate that NeuroD1 can reprogram reactive astrocytes into functional neurons in the dorsal horn of the injured spinal cord.

### NeuroD1-Mediated Cell Conversion in the Contusive SCI Model

**Figure 7:**
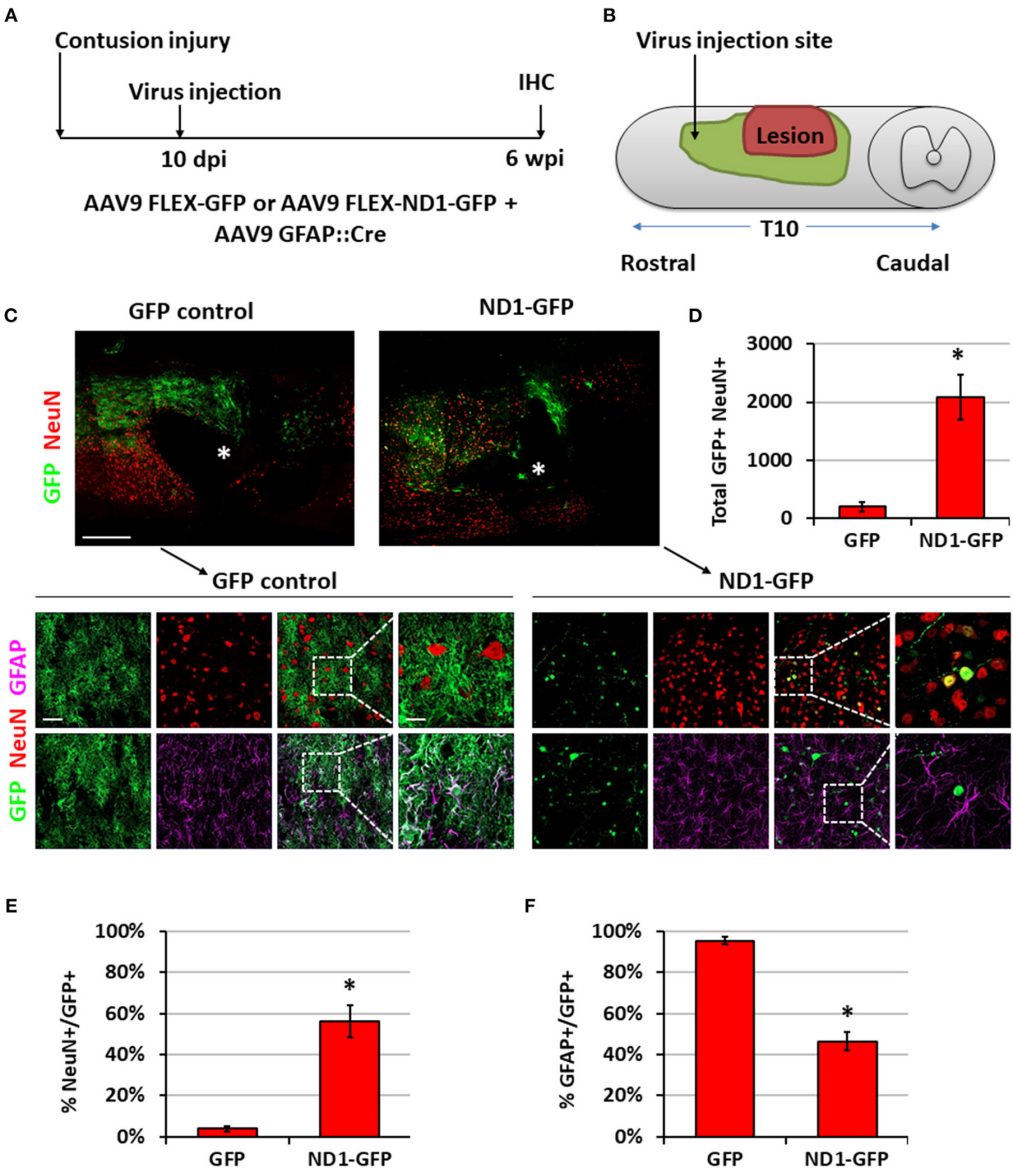
NeuroD1 converts reactive astrocytes into neurons around the injury core with a short delay of viral injection after contusive SCI. **(A)** Experimental design showing viral injection at 10 days post a contusive SCI (30 Kdyn force). Spinal cords were analyzed at 6 weeks post viral infection. **(B)** Schematic drawing shows viral injection position: 1 mm anterior of the contusion site, 0.4 mm lateral of the central artery, and from 0.8 mm to 0.4 mm below the tissue surface. **(C)** Many infected cells survived around the injury core (indicated by *) and showing distinct cellular morphology between the two groups. Immunostaining of the neuronal markers GFAP and NeuN indicates successful neuronal conversion from reactive astrocytes by NeuroD1-GFP. Scale bars, 1 mm at overview, 50 μm at low-mag, 20 μm at high-mag. **(D)** Quantified number of converted neurons per infection (i.e., the average number of both GFP^+^ and NeuN^+^ cells per horizontal section calculated from one dorsal, one central, and one ventral section, and then multiplied by the total number of horizontal sections per sample). Kruskal-Wallis *H*-test shows a significant difference between the two groups (*n* = 3 for each group; *p* = 0.05). **(E)** Quantified NeuN^+^ signal at 6 wpi. Kruskal-Wallis *H*-test shows a significant difference between the two groups (*n* = 3 for each group; *p* = 0.05). **(F)** GFAP staining shows a significant decrease in the number of astrocytes after NeuroD1 conversion at 6 wpi. Kruskal-Wallis *H*-test shows a significant difference between the two groups (*n* = 3 for each group; *p* = 0.05).

**Figure 8:**
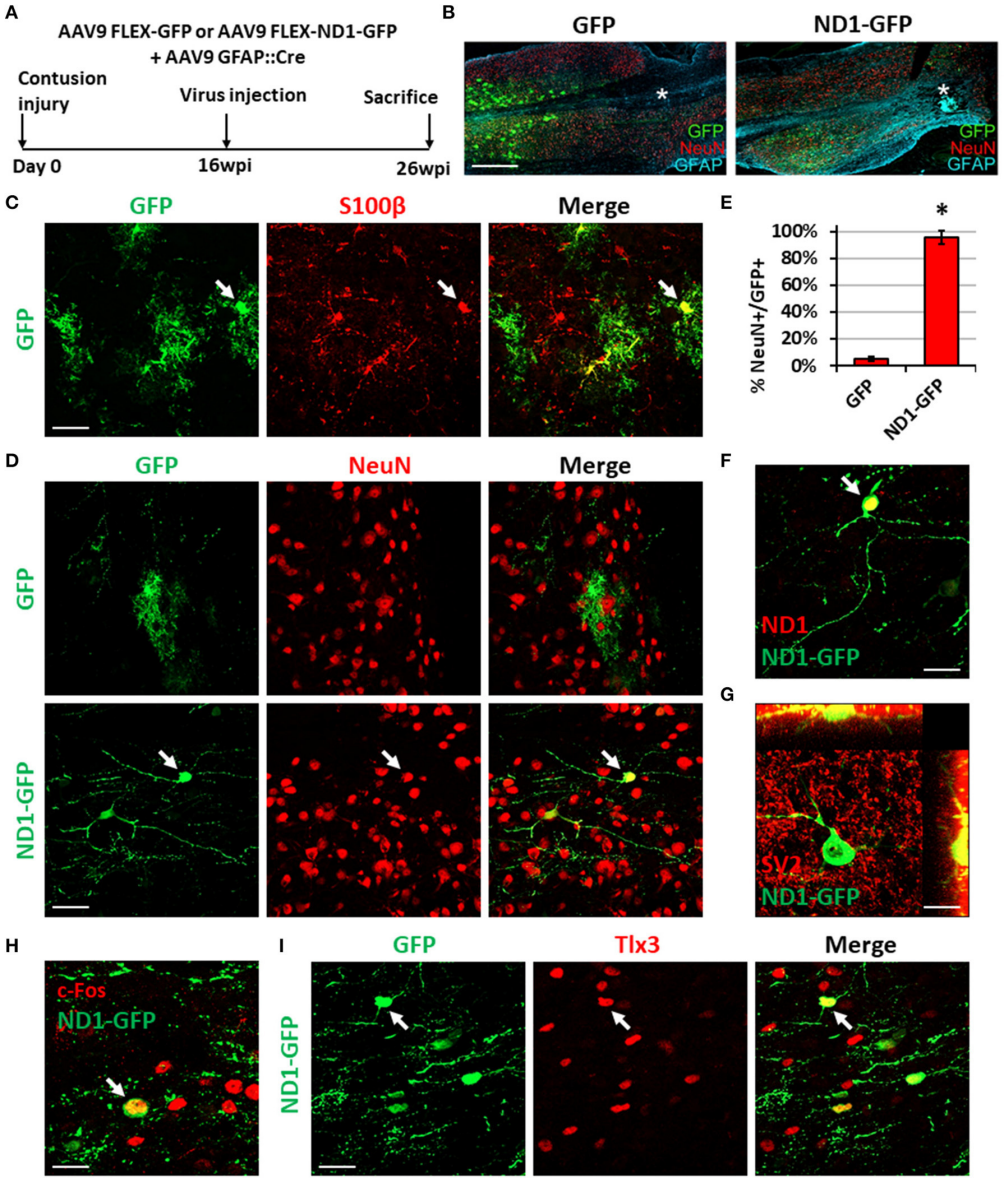
NeuroD1-mediated neuronal conversion with a long delay of viral injection after contusive SCI. **(A)** Experimental design showing viral injection at 16 weeks after a contusive SCI (30 Kdyn force). The spinal cords were analyzed at 10 weeks post viral infection. **(B)** Many infected cells survived around the injury core (indicated by *) and showing distinct cellular morphology between the two groups. Scale bar, 1 mm. **(C)** Co-expression of the astrocyte marker S100b in control AAV GFP-infected cells. Scale bar, 50 μm. **(D)** Immunostaining of the neuronal marker NeuN indicates successful neuronal conversion from reactive astrocytes by NeuroD1-GFP with high efficiency. Scale bar, 50 μm. **(E)** Quantified neuronal conversion efficiency (95.6 ± 5.1%) at 10 wpi. Kruskal-Wallis *H*-test shows a significant difference between the two groups (*n* = 4 for the NeuroD1-GFP group and *n* = 3 for the GFP group; *p* = 0.034). **(F)** Co-expression of NeuroD1 protein in the NeuroD1-GFP-infected cells. Scale bar, 20 μm. **(G)** Co-expression of synaptic marker SV2 on the NeuroD1-GFP^+^ cells. **(H)** Co-expression of the neuronal activity marker c-Fos in the NeuroD1-GFP^+^ cells. Scale bar, 20 μm. **(I)** Co-expression of the glutamatergic subtype marker Tlx3 in NeuroD1-GFP^+^ neurons in the spinal cord dorsal horn. Arrows show an example Tlx3^+^ cell. Scale bar, 20 μm.

To move closer toward clinical situations, we evaluated NeuroD1-mediated neuronal conversion in the contusive SCI model. Compared with stab injury, contusive injury creates a much more severe injury environment, which could affect the efficiency of neuronal conversion and the survival of converted neurons. We therefore performed two experiments to test our AAV GFAP::Cre and Flex-NeuroD1-GFP system after contusive SCI: one short-delay injection to test our treatment as a response to acute injury (Figure 7) and one long-delay injection to test our treatment as a response to chronic injury (Figure 8). The advantage of the short-delay experiment is to maximize infection rate by taking advantage of the post-injury proliferation of reactive astrocytes, while the advantage of the long-delay experiment is to maximize the neuronal survival after conversion by allowing injury-induced neuroinflammation to taper down and minimize the secondary effects of the contusion injury. In our short-delay experiment, viral injection was conducted at 10 days post-contusive injury and tissues were collected at 6 weeks post-viral infection (Figure 7A). Viral injections were performed 1 mm away from the contusion site to avoid the injury core (Figure 7B). The injury core is apparent after contusion and is characterized by the loss of NeuN^+^ neuronal cell bodies (Figure 7C, labeled by ^*^). Viral injection at 10 days post-contusion resulted in many GFP^+^ cells surrounding the injury core in both control GFP and NeuroD1-GFP groups (Figure 7C), indicating good infection rate and survival of the AAV-infected cells in the contusive SCI model. On the other hand, the AAV NeuroD1-GFP infected cells showed a dramatic morphological difference from the control GFP group (Figure 7C). As illustrated in the enlarged images in Figure 7C, the GFP infected cells in the control group showed typical astrocytic morphology and colocalization with GFAP signal (magenta), but rarely showed any colocalization with the neuronal marker NeuN (red). In contrast, NeuroD1-GFP infected cells were often colocalized with NeuN but rarely colocalized with GFAP (Figure 7C), indicating successful neuronal conversion. Quantitatively, we counted the total number of converted neurons to be ~2,000 cells surrounding the lesion core areas (Figure 7D). The efficiency of NeuroD1-mediated neuronal conversion in the short-delay experiment as measured by NeuN immunoreactivity was ~55% (Figure 7E), while the remaining cells were mostly GFAP^+^ (Figure 7F). In contrast, the GFP-infected cells were mostly GFAP^+^ astrocytes and rarely NeuN^+^ neurons (only 3.9% NeuN^+^ in GFP group) (Figures 7E,F).

In our long-delay experiment, viral injection was conducted at 4 months post-contusive injury, when glial scar has been well formed after contusion, and tissues were collected at 10 weeks post-viral infection (Figure 8A). As in the short-delay experiment, the injury core was apparent after contusion and characterized by the loss of NeuN^+^ signal (Figure 8B, labeled by ^*^). In the control AAV GFP alone group, the viral infected cells were mainly S100b^+^ astrocytes (Figure 8C), but rarely showed any NeuN^+^ signal (Figure 8D, top row). In contrast, the majority of NeuroD1-GFP infected cells were converted into NeuN^+^ neurons (Figure 8D, bottom row; quantified in Figure 8E). The NeuroD1-mediated conversion efficiency reached >95% (Figure 8E). We observed mature neuronal morphology including longer and more branching processes at this late time point and confirmed NeuroD1 overexpression in the NeuroD1-GFP infected cells (Figure 8F). Furthermore, NeuroD1-converted neurons at 10 wpi were surrounded by many synaptic puncta (SV2) with some of them directly innervating the soma and dendrites (Figure 8G, yellow dots). We also identified c-Fos^+^ cells among NeuroD1-converted neurons (Figure 8H), indicating that they were able to integrate into the local spinal cord functional circuitry. Lastly, some of the NeuroD1-converted neurons in the contusive SCI model at 10 wpi showed glutamatergic subtype through expression of Tlx3 in the dorsal horn (Figure 8I), consistent with our stab injury model. Altogether, these results indicate that NeuroD1 overexpression can reprogram reactive astrocytes into functional neurons after contusive SCI under both acute and chronic treatment conditions, with higher conversion efficiency achieved after glial scar formation. This clinically relevant model can be used in future studies to further test functional improvement after SCI using *in vivo* cell conversion technology.

## Discussion

In this study, we have demonstrated in different SCI models that overexpression of NeuroD1 in the reactive astrocytes can convert them into Tlx3-positive glutamatergic neurons in the dorsal horn of injured spinal cord. Other ongoing studies are also testing different transcription factors to reprogram spinal astrocytes into GABAergic neurons or motor neurons in the ventral horn. Using AAV Cre-Flex system, we efficiently reprogrammed reactive astrocytes after stab injury in the dorsal horn of spinal cord into functional neurons that integrate into the local synaptic network. Importantly, we also observed efficient NeuroD1-mediated neuronal conversion in a contusive SCI model making this technique a potential intervention to treat SCI by regenerating functional new neurons in the gray matter. The NeuroD1-converted neurons in the dorsal horn of injured spinal cord were Tlx3^+^ glutamatergic spinal neurons, but not Tbr1^+^ cortical neurons, indicating regional specificity of *in vivo* reprogramming. The fact that the same neural transcription factor NeuroD1 can convert cortical astrocytes into cortical neurons and spinal astrocytes into spinal neurons may hold the key to region-specific neural repair by using internal glial cells for neuroregeneration. In contrast, transplantation of external cells does not have this advantage of intrinsic cell lineage with region-specificity.

### AAV Gene Delivery System for Neuronal Conversion

We have accomplished successful NeuroD1-mediated neuronal conversion with retroviral vectors in this study and previous studies (Guo et al., 2014; Chen et al., 2020). However, since retrovirus particles are relatively large, limiting the viral titer, and since retrovirus only infects proliferating cells, the viral injection timing is confined to a narrow time window after the injury during which glial cell proliferation is increased. In contrast, because AAV infects both proliferating and quiescent cells, the AAV injection depends less on timing and can be performed in both acute and chronic injury conditions. Additionally, AAV has passed clinical trials and elicits little immune response when applied *in vivo* (Zaiss et al., 2002; High and Roncarolo, 2019). AAV particles are small and can be prepared at very high titer, therefore high dosage of conversion factors can be achieved when multiple AAV particles infect a single cell. Consistent with this, we observed a slower neuronal conversion when a lower titer of AAV-NeuroD1 was injected in the injured spinal cord (unpublished observation). Since certain serotypes of AAV can cross the blood-brain-barrier (BBB), intravenous delivery of conversion factors has made it possible to reach a broader area of the CNS (Foust et al., 2009; Chan et al., 2017). Following our earlier report of efficient conversion of reactive astrocytes into functional neurons by NeuroD1 through retroviral infection (Guo et al., 2014), intravenous injection of AAV9 expressing NeuroD1 has been reported to infect a small but significant number of resting astrocytes in the striatum and convert them into neurons (Brulet et al., 2017). On the other hand, our intracranial injection of AAV9 expressing NeuroD1 in stab injury model (Zhang et al., 2020) and ischemic injury model (Chen et al., 2020) have both resulted in high conversion efficiency, suggesting that reactive astrocytes after injury are more likely converted into neurons than the resting astrocytes. Consistent with this hypothesis, our results in this study also support that reactive astrocytes in injured spinal cord can be effectively converted into neurons with ~95% efficiency, regardless of retrovirus or AAV delivery system. Compared to the high efficiency of astrocyte-converted neurons in the NeuroD1 group, we did observe a small percentage (typically <5%) of neurons labeled by GFP in the control group (Figure 8E). This might be due to the fact that AAV can infect both neurons and astrocytes efficiently. While we have used GFAP promoter to restrict the transgene expression in astrocytes, it is known that promoters usually are not 100% specific, and GFAP promoter can have low activity in neurons especially under injury condition.

The high efficiency of astrocyte-to-neuron conversion is the major reason why we target astrocytes among glial cells for *in vivo* conversion. Importantly, we have demonstrated in the mouse cortex that after astrocyte-to-neuron conversion, the remaining astrocytes can proliferate and replenish themselves (Zhang et al., 2020). Unlike killing reactive astrocytes which has been reported to make injury worse (Anderson et al., 2016), we demonstrated that converting reactive astrocytes into functional new neurons significantly ameliorated the glial scar in the brain, leading to a reversal of glial scar back to neural tissue (Zhang et al., 2020). Another practical reason for targeting astrocytes is that among glial cells including astrocytes, NG2 cells (OPCs), and microglia, AAV preferentially infect astrocytes, with much less infection rate on NG2 cells or microglia. AAV-based gene therapy so far is relatively safe and preferred choice for CNS disorders, and FDA has approved a series of clinical trials using AAV. In order to develop AAV-based gene therapy for the treatment of CNS disorders, we selected astrocytes as our preferred target for *in vivo* cell conversion. Of course, other glial cells such as NG2 cells and microglia might have their own advantage for some particular purpose (Guo et al., 2014; Heinrich et al., 2014; Torper et al., 2015; Pereira et al., 2017; Matsuda et al., 2019).

### Distinct Functions of NeuroD1 During Neuronal Conversion

Neuronal conversion can be achieved by several neurogenic transcription factors (Li and Chen, 2016). Besides NeuroD1, Sox2, Ngn2, and Ascl1 have all been reported to convert glial cells into neurons (Grande et al., 2013; Niu et al., 2013; Guo et al., 2014; Heinrich et al., 2014; Su et al., 2014; Liu et al., 2015; Gascon et al., 2016). Sox2 is expressed in neural progenitors and functions to maintain progenitor identity (Bylund et al., 2003; Graham et al., 2003; Bani-Yaghoub et al., 2006). Therefore, it is not surprising that Sox2-mediated neuronal conversion has to go through a proliferation stage as shown by incorporation of BrdU and the expression of Ki67 (Su et al., 2014). In contrast, NeuroD1 is a neuronal differentiation transcription factor that instructs terminal differentiation of neuroprogenitors into neurons during early neural development (Miyata et al., 1999; Morrow et al., 1999; Gao et al., 2009). This might partially explain why NeuroD1 can achieve high neuronal conversion efficiency (>90%) comparing to ~6% in the case of Sox2 (Su et al., 2014). An earlier report also showed that Ngn2-expressing retrovirus was able to promote neurogenesis in the injured spinal cord, but the number of newly generated neurons greatly decreases over time even when combined with growth factor treatment (Ohori et al., 2006). When combined with Bcl2, an anti-apoptotic gene, Ngn2-mediated neuronal conversion acquires a much higher efficiency, although the authors claim that Bcl2 plays a role independent of apoptotic pathways (Gascon et al., 2016). In sharp contrast, we rarely observe apoptotic cells during and after NeuroD1-mediated conversion as determined by TUNEL assay. The difference of cell survival in converted neurons between different transcription factors may be explained by the fact that NeuroD1 is not only a conversion factor but also a survival factor. During development, NeuroD1 is required for survival of a variety of neuronal subtypes in the developing and adult CNS (Miyata et al., 1999; Morrow et al., 1999; Gao et al., 2009). This dual role of NeuroD1 during neuronal conversion and neuronal survival may explain its higher conversion efficiency over Sox2 and Ngn2. Ascl1 has also been reported to induce high efficiency of *in vivo* astrocyte-to-neuron conversion in the midbrain (Liu et al., 2015), suggesting that Ascl1 might share certain common properties with NeuroD1. Since long-term survival of converted neurons is essential to their integration into local neuronal circuitry in order to have a role in functional repair, future studies on clinical translation must pay much attention to the total number of newly generated neurons that can survive for years to have effective therapies.

### Environmental Cues to Impact Neuronal Conversion in Addition to Intrinsic Factors

Transcription factor-mediated *in vivo* neuronal reprogramming illustrates intrinsic power to convert reactive astrocytes into neurons. However, environmental cues also play a role in the success of neuronal conversion (Heinrich et al., 2014) as well as the phenotype of converted neurons (Grande et al., 2013). In this study, we found that NeuroD1-converted neurons in the injured mouse cortex were Tbr1^+^ cortical neurons, but in the injured spinal cord the NeuroD1-converted neurons were Tlx3^+^ spinal neurons. Therefore, the local environment, together with astroglial lineage, may be essential to functional integration of converted neurons into the local neuronal circuitry as they mature. Together, a complete neuronal conversion would need both intrinsic factors (transcription factors and glial lineage factors) and extrinsic factors (local cues) to solidify the identity of converted neurons.

Even within the spinal cord, local environment can be drastically different between the gray matter vs. the white matter, with neuronal soma confined to the gray matter and neuronal axons occupying the white matter. In our experiments using AAV, we rarely observed converted neurons in the white matter; using retrovirus, we observed some converted neurons in the white matter at early time points, but they rarely survived to 6 wpi. This has been similarly observed in cell conversion studies in the white matter (corpus callosum) of mouse brains (Liu et al., 2020). Reasons for the lack of conversion in the white matter in this study could include our targeted injection technique which delivers virus precisely into the dorsal horn of the gray matter or the lack of appropriate viral receptors in the white matter. White matter may also lack sufficient trophic factors for the survival of newly generated neurons. On the other hand, Sox2-mediated neuronal conversion can result in many newborn neurons located in the white matter of the spinal cord, particularly in p21 knockout mice (Wang et al., 2016). An interesting feature of these neurons is that they appear as clusters, which, by providing trophic factors to each other, could be the reason they survive. It is also possible that the local environment in the white matter of p21 knockout mice has been altered during Sox2-mediated neuronal conversion in combination with BDNF and noggin. Further studies will be required to determine the differential effects of not only gray matter vs. white matter but also dorsal horn vs. ventral horn on neuronal conversion.

### Generation of Neuronal Subtypes via NeuroD1-Mediated Conversion

We demonstrate here that NeuroD1 converts reactive astrocytes into primarily Tlx3-positive glutamatergic neurons in the dorsal horn of the injured spinal cord. Interestingly, Sox2-converted neurons in the injured spinal cord are also mainly glutamatergic (Wang et al., 2016), raising the possibility that glutamatergic neurons might be a default subtype of converted neurons. On the other hand, when we combine NeuroD1 with Dlx2 together, the proportion of GABAergic neurons was significantly increased after conversion, suggesting that the composition of neural transcription factors play an important role in the fate determination after conversion. NeuroD1 overexpression itself has been shown to inhibit GABAergic neuronal differentiation by suppressing Ascl1 (Mash1) (Roybon et al., 2010). Our result of NeuroD1 + Dlx2 suggests that Dlx2 can at least partially antagonize the effect of NeuroD1 and pushing the astrocyte conversion more toward GABAergic neurons.

We noted that the glutamatergic neurons in the dorsal horn of the spinal cord can be Tlx3^+^, BarH1^+^, or FoxD3^+^ (Bermingham et al., 2001; Gross et al., 2002; Cheng et al., 2005; reviewed in Lu et al., 2015). Therefore, some of the non-identified neurons may include BarH1^+^ or FoxD3^+^ glutamatergic neurons. On the other hand, considering the newly generated neurons were converted from astrocytes, they may not be mature enough to acquire definitive neuronal identity yet, or perhaps some will not become the neurons similar to their neighbors. More studies are required to fully characterize the neuronal identity after conversion for much longer time, such as 4–6 months after viral infection.

## Conclusion

In summary, our study demonstrates that AAV NeuroD1-based gene therapy can convert reactive astrocytes into functional new neurons with high efficiency in the injured spinal cord. The AAV-NeuroD1 converted neurons can functionally mature and integrate into local neural networks. Interestingly, NeuroD1-converted neurons in the spinal cord dorsal horn mainly acquire a Tlx3^+^ glutamatergic neuronal subtype, while combining NeuroD1 and Dlx2 together can generate more GABAergic neurons. Our next challenge is to further identify transcription factors that can regenerate motor neurons in the ventral horn, and ultimately test the beneficial effects of this cutting-edge *in vivo* astrocyte-to-neuron conversion technology in spinal cord repair.

## Data Availability Statement

The raw data supporting the conclusions of this article will be made available by the authors, without undue reservation.

## Ethics Statement

The animal study was reviewed and approved by Penn State IACUC.

## Author Contributions

GC conceived the idea, supervised the entire project, discussed the results, analyzed the data, and revised and finalized the manuscript. HL helped with the supervision of the project, performed some surgery experiment, discussed and analyzed the result, and helped with the manuscript writing. BP performed the major experiments, analyzed the data, made the figures, and wrote the draft of the manuscript. YD performed the contusive SCI experiments with the help from HL. BP analyzed the figure, made the figures, and wrote part of the manuscript. All other authors helped with the experiments and analyzed the data.

## Conflict of Interest

GC is a co-founder of NeuExcell Therapeutics Inc. The remaining authors declare that the research was conducted in the absence of any commercial or financial relationships that could be construed as a potential conflict of interest.
