# Supplementary material for: Regeneration of Functional Neurons After Spinal Cord Injury via in situ NeuroD1-Mediated Astrocyte-to-Neuron Conversion
Source: Front Cell Dev Biol. 2020 Dec 16;8:591883. doi: 10.3389/fcell.2020.591883 (PMC7793709; doi:10.3389/fcell.2020.591883)
Supplement: Supplementary file 1 [file Data_Sheet_1.docx]

**Supplementary Material**

**Regeneration of functional neurons after spinal cord injury via *in situ* NeuroD1-mediated astrocyte-to-neuron conversion**

Brendan Puls^1^†, Yan Ding^1^†, Fengyu Zhang^1^, Mengjie Pan^1^, Zhuofan Lei^1^, Zifei Pei^1^, Mei Jiang^1^, Yuting Bai^1^, Cody Forsyth^1^, Morgan Metzger^1^, Tanvi Rana^1^, Lei Zhang^1^, Xiaoyun Ding^1^, Matthew Keefe^1^, Alice Cai^1^, Austin Redilla^1^, Michael Lai^1^, Kevin He^1^, Hedong Li^1,^*, Gong Chen^1,2,^*

**Supplemental figures and legends:**


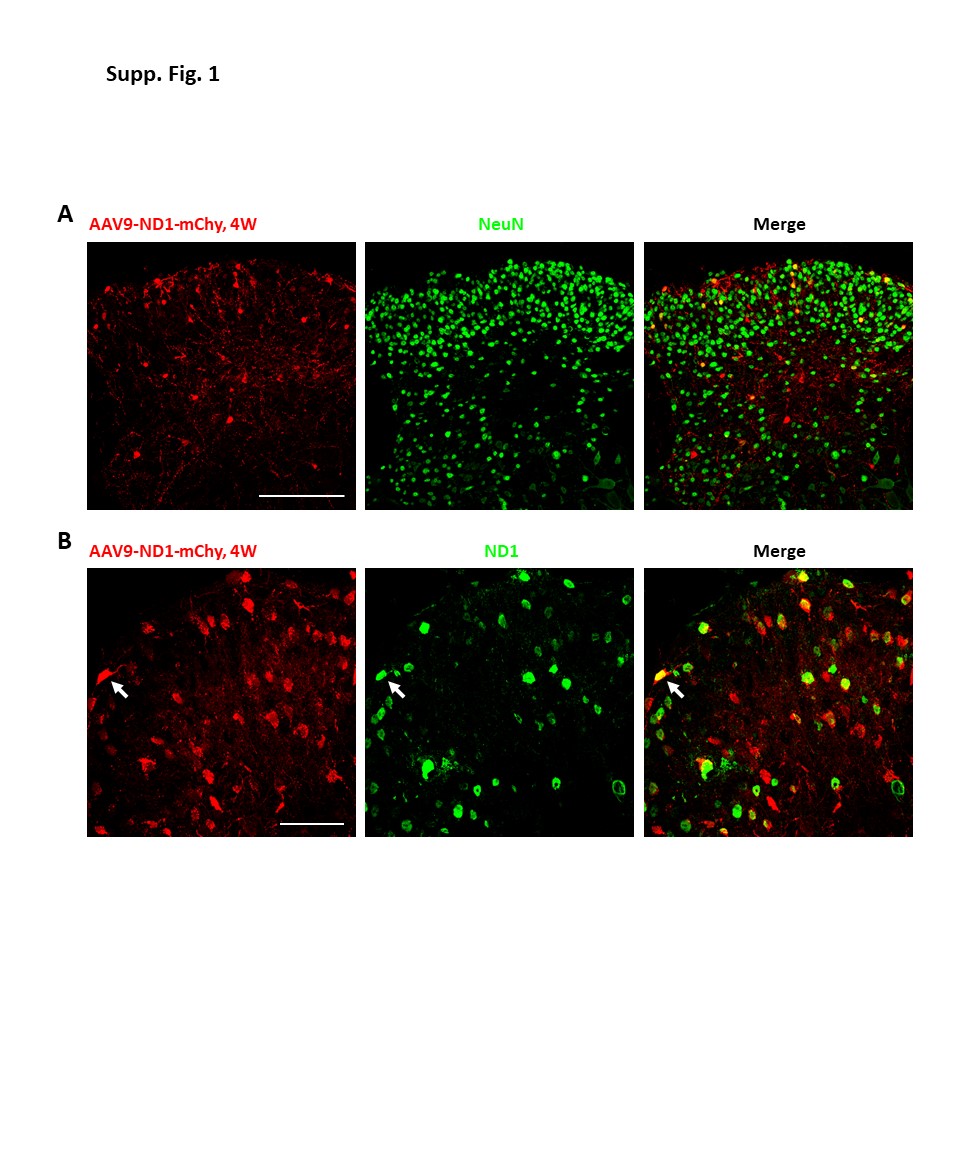


**Supplemental Figure 1. Infected cells by AAV9-NeuroD1-mCherry overexpress NeuroD1 protein in the injured spinal cord.** (**A**) Immunostaining showing the colocalization of infected cells by AAV9 NeuroD1-mCherry with neuronal marker NeuN, indicating neuronal conversion in the dorsal horn of injured spinal cord at 4 wpi. Scale bar, 200 µm. (**B**) Infected cells overexpressed NeuroD1 protein at 4 wpi. Scale bar, 50 µm.


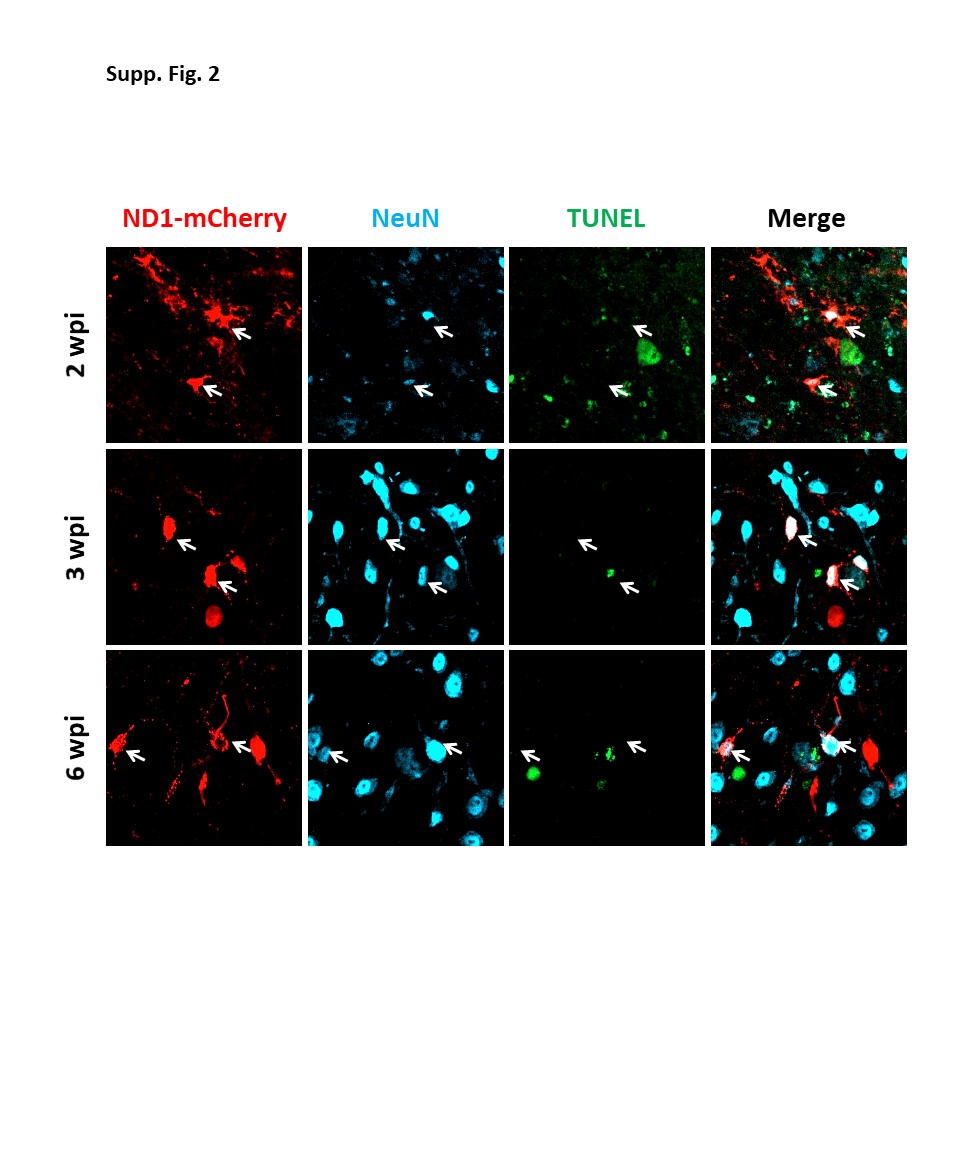


**Supplemental Figure 2. NeuroD1-mediated neuronal conversion does not involve severe apoptosis.** TUNEL staining was performed to detect apoptotic cells at different stages of neuronal conversion by AAV9 NeuroD1-mCherry in the injured spinal cord. Arrows show infected cells that are NeuN-positive but TUNEL−negative.


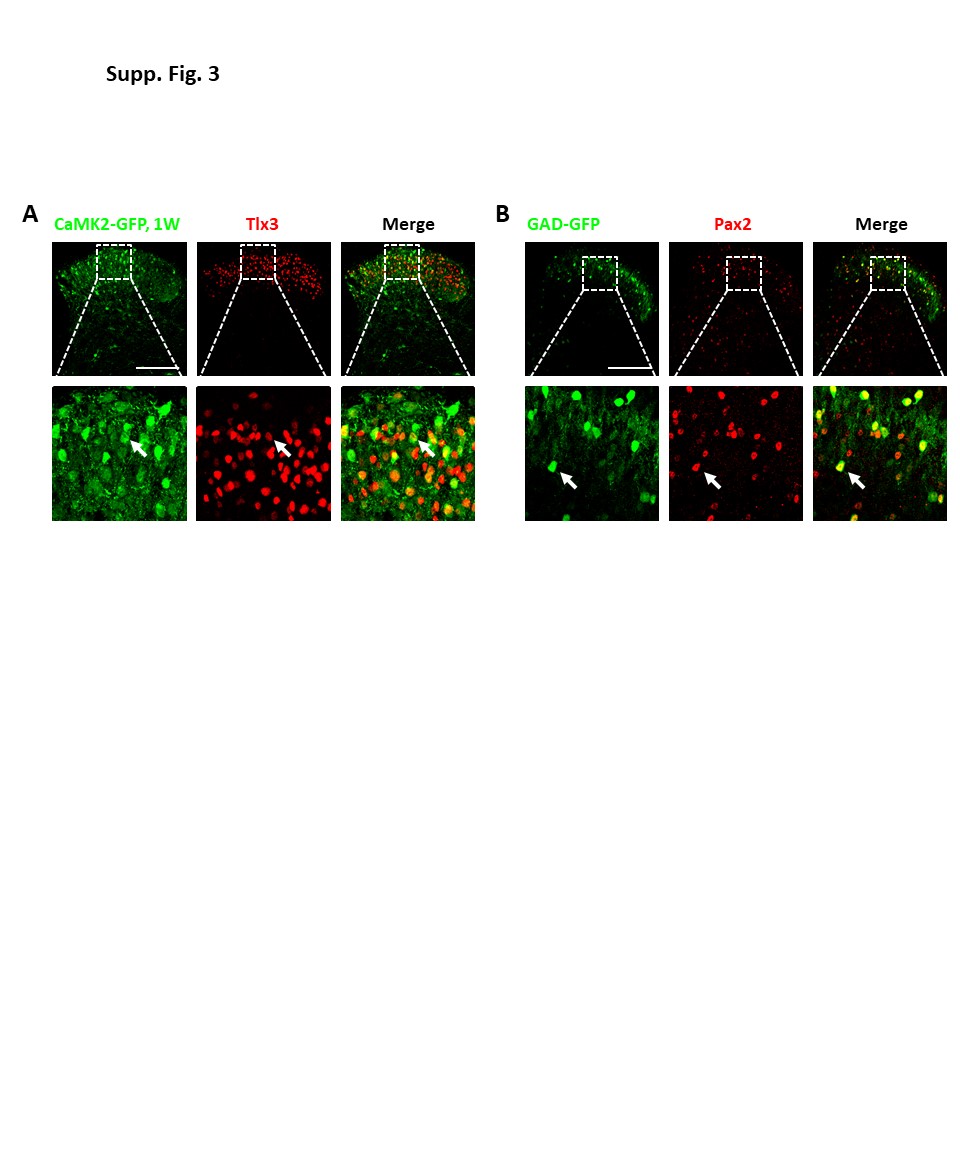


**Supplemental Figure 3. CaMK2-GFP virus and GAD-GFP mice can be used to confirm neuronal subtype.** (**A**) AAV9 CaMK2-GFP-infected cells co-stains with Tlx3. (**B**) GAD-GFP transgenic mouse shows co-staining of GAD-GFP with Pax2. These data indicate that CaMK2-GFP and GAD-GFP can be used to confirm glutamatergic and GABAergic subtypes in the spinal cord. Scale bar, 200 µm.
